# An RIG-I-Like RNA Helicase Mediates Antiviral RNAi Downstream of Viral siRNA Biogenesis in *Caenorhabditis elegans*

**DOI:** 10.1371/journal.ppat.1000286

**Published:** 2009-02-06

**Authors:** Rui Lu, Erbay Yigit, Wan-Xiang Li, Shou-Wei Ding

**Affiliations:** 1 Department of Plant Pathology & Microbiology, University of California, Riverside, California, United States of America; 2 Program in Molecular Medicine, University of Massachusetts Medical School, Worcester, Massachusetts, United States of America; Stanford University, United States of America

## Abstract

Dicer ribonucleases of plants and invertebrate animals including *Caenorhabditis elegans* recognize and process a viral RNA trigger into virus-derived small interfering RNAs (siRNAs) to guide specific viral immunity by Argonaute-dependent RNA interference (RNAi). *C. elegans* also encodes three *Dicer-related helicase* (*drh*) genes closely related to the RIG-I-like RNA helicase receptors which initiate broad-spectrum innate immunity against RNA viruses in mammals. Here we developed a transgenic *C. elegans* strain that expressed intense green fluorescence from a chromosomally integrated flock house virus replicon only after knockdown or knockout of a gene required for antiviral RNAi. Use of the reporter nematode strain in a feeding RNAi screen identified *drh-1* as an essential component of the antiviral RNAi pathway. However, RNAi induced by either exogenous dsRNA or the viral replicon was enhanced in *drh-2* mutant nematodes, whereas exogenous RNAi was essentially unaltered in *drh-1* mutant nematodes, indicating that exogenous and antiviral RNAi pathways are genetically distinct. Genetic epistatic analysis shows that *drh-1* acts downstream of virus sensing and viral siRNA biogenesis to mediate specific antiviral RNAi. Notably, we found that two members of the substantially expanded subfamily of Argonautes specific to *C. elegans* control parallel antiviral RNAi pathways. These findings demonstrate both conserved and unique strategies of *C. elegans* in antiviral defense.

## Author Summary

The genome of *Caenorhabditis elegans* encodes three Dicer-related helicases (DRHs) highly homologous to the DExD/H box helicase domain found in two distinct families of virus sensors, Dicer ribonucleases and RIG-I-like helicases (RLRs). Dicer initiates the specific, RNAi-mediated viral immunity in plants, fungi and invertebrates by producing virus-derived small interfering RNAs (siRNAs). By contrast, mammalian RLRs trigger interferon production and broad-spectrum viral immunity, although one of the three RLRs may act as both a negative and positive regulator of viral immunity. In this study we developed a transgenic *C. elegans* strain for high-throughput genetic screens and identified 35 genes including *drh-1* that are required for RNAi-mediated viral immunity. Genetic epistatic analyses demonstrate that *drh-1* mediates RNAi immunity downstream of the production of viral siRNAs. Notably, we found that *drh-2* functions as a negative regulator of the viral immunity. Thus, both nematode DRHs and mammalian RLRs participate in antiviral immune responses. Unlike mammalian RLRs, however, nematode DRH-1 employs an RNAi effector mechanism and is unlikely to be involved in direct virus sensing.

## Introduction

Innate immunity is active immediately upon pathogen attack and represents an ancient defense mechanism conserved in diverse multicellular organisms. Innate immunity is initiated by pattern recognition receptors (PRRs) that recognize conserved molecular patterns associated with microbes. Well-characterized PRR families include the transmembrane Toll-like receptors (TLRs) and the cytosolic NOD-like receptors (NLRs) and RIG-I-like RNA helicase receptors (RLRs), all of which contain members in vertebrates that recognize viral single- and/or double-stranded RNAs as the pathogen signatures 1–3. Recognition of pathogens by PRRs typically triggers protein-protein interactions of PRRs with downstream signaling factors leading to the nucleus translocation of a transcriptional factor such as NF-κB and the subsequent transcription of immunity effector genes. The Dicer family of ribonucleases also recognizes viral RNA like these PRRs to initiate the viral immunity in plants and invertebrates that is mechanistically related to RNA silencing or RNA interference (RNAi). Unlike TLR and RLRs, however, Dicer further processes the viral RNA trigger into small RNAs of 21–24 nucleotides to guide specific antiviral silencing [4].

In addition to two type III RNase domains and a dsRNA-binding domain (dsRBD), Dicer contains an RNA binding domain called PAZ and an N-terminal RNA helicase domain that is closely related to RLRs [5],[6]. The Dicer family proteins produce small interfering RNAs (siRNAs) and microRNAs (miRNAs) in many eukaryotes, which are loaded in an Argonaute (AGO)-containing effector complex to silence gene expression by RNA cleavage, translational arrest, or methylation of DNA and chromatin. In fungi, plants and *Caenorhabditis elegans*, siRNAs are further amplified in a process that depends on de novo synthesis of dsRNA by cellular RNA-dependent RNA polymerase (RDR).

Genetic requirements of the Dicer-initiated viral immunity have been characterized more extensively in *Arabidopsis thaliana* and *Drosophila melanogaster* using known mutants in various RNA silencing pathways [7]. The prevailing model for antiviral silencing against RNA viruses is that it acts via the canonical dsRNA-siRNA pathway of RNAi. This is supported by the detection of virus-derived siRNAs (viRNAs) of two polarities covering the entire length of viral genomic RNAs in the infected cells and the identification of the siRNA-producing Dicers in the biogenesis of viRNAs in both *D. melanogaster* and *A. thaliana* [4], [8]–[14]. Almost all of the genes known to participate in *A. thaliana* antiviral silencing have been implicated in the RDR-dependent synthesis of dsRNA in transgene-induced RNA silencing [4], [15]–[20]. In *D. melanogaster*, antiviral silencing induced by distinct positive-strand RNA viruses including Flock house virus (FHV), requires the same set of the core components of the canonical RNAi pathway that are dispensable for the biogenesis and function of miRNAs [13],[14],[21],[22]. A recent study found that viral dsRNA produced during initiation of FHV progeny RNA synthesis is recognized by Dicer-2 (DCR-2) and diced into viRNAs to trigger antiviral silencing [23]. Infection of mammalian cells with some DNA viruses induces production of virus-derived miRNAs capable of silencing the antisense mRNAs of the cognate viruses [24]. However, there is currently no evidence for the production of viral siRNA in mammals in response to RNA viruses, suggesting that RNA viruses are sensed by unrelated PRRs in invertebrates and vertebrates [4].

*C. elegans* is an excellent model system for studying many aspects of biology, including host responses to bacterial pathogens [25],[26]. *C. elegans* lacks NLRs and NF-κB-like transcriptional factors but encodes a single TLR. *C. elegans* also encodes a family of Dicer-related helicases (DRH), DRH-1, DRH-2 and DRH-3, which are highly homologous to the DExD/H box RNA helicase domain found in Dicer and the mammalian RLR family composed of RIG-I, MDA5 and LGP2 [6],[27],[28]. The RNA silencing machinery of *C. elegans* is characterized by a single Dicer (*dcr-1*), 4 RDRs (eg, *ego-1* and *rrf1*–*rrf-3*) and 27 AGOs [29],[30]. Both miRNAs and siRNAs are produced by DCR-1 whereas PIWI-interacting RNAs (piRNAs, also known 21U RNAs) is Dicer-independent. Processing both endogenous (endo) and exogenous (exo) dsRNA into siRNAs further requires the dsRNA-binding protein RDE-4 although distinct AGO and RDR proteins participate in endo and exo siRNA pathways [6], [31]–[35]. The *C. elegans* family of AGOs, the largest of any organisms examined to date, is divided into three subfamilies. The AGO and PIWI subfamilies are required for the biogenesis of miRNAs and piRNAs, respectively, but *ergo-1* in the PIWI subfamily has an essential role in the production of endo-siRNAs [31], [36]–[38]. The third subfamily is worm-specific and contains 18 members, many of which such as *rde-1*, *ppw-1*, *C04F12*.1, *sago-1*, and *csr-1*, act in parallel or sequentially to mediate the exo-siRNA pathway [39]–[42]. The exo-siRNA pathway requires amplification by *rrf-1* in the soma and *ego-1* in the germline whereas *rrf-3* is essential for the biogenesis of endo-siRNAs [34],[43]. Interestingly, exo-RNAi is enhanced in worm mutants defective for several components of the endo-siRNA pathway including *eri-1*, *ergo-1* and *rrf-3*, suggesting antagonism between the two siRNA pathways [27],[30],[34],[41],[44],[45].

A natural virus for *C. elegans* is not known. However, cultured primary cells and living animals of *C. elegans* can be infected respectively by Vesicular stomatitis virus (VSV) and Vaccinia virus and living animals support complete replication of the FHV RNA genome engineered to be transcribed from an integrated transgene [46]–[49]. Infection of VSV, which contains a negative-strand RNA genome, is associated with the production of VSV-specific small RNAs and is potentiated in both nematode cells derived from exoRNAi-defective *C. elegans* mutants (*rde-1*; *rde-3*, *rde-4*, and *rrf-1*) and wild-type cells depleted of either DCR-1 or C04F12.1, but is inhibited in *rrf-3* and *eri-1* mutants that exhibit an enhanced exoRNAi response [47],[48]. The positive-strand genome of FHV is divided into two RNAs. RNA1 (3.1 kb) encode the viral RNA-dependent RNA polymerase (RdRP) and can self-replicate independently of RNA2 (1.4 kb), which encodes the capsid protein. Although FHV is a natural insect virus, it replicates efficiently on outer mitochondria membranes of diverse eukaryotic cells, indicating conserved mitochondria targeting of the viral replication complex [50]–[52]. FHV RNA3 (387 nt) is a product of RNA1 replication and acts as mRNA of the B2 protein, a viral suppressor of RNAi (VSR) essential for FHV infection in *D. melanogaster* [13],[14],[21],[53]. We showed that replication of a B2-deficient FHV mutant occurred robustly in the *rde-1* mutant nematodes but was severely inhibited in wild-type nematodes, indicating restriction of FHV replication by the exo-RNAi pathway in *C. elegans* [46]. These studies together strongly indicate that *C. elegans* encodes an active antiviral RNAi pathway that is induced by either direct viral infection [47],[48] or replication of a viral RNA genome initiated intracellularly [46], the latter of which bypasses the initial steps such as cell entry that occur in viral infection of natural hosts.

Here we describe development of a transgenic *C. elegans* strain for the genetic characterization of the antiviral RNAi pathway. Use of the reporter strain in a feeding RNAi screen led to the identification of a largest set of putative genes in antiviral RNAi pathway in any organism. In particular, we showed that *drh-1* and *drh-2* of the three nematode *drh* genes participated in the regulation of antiviral RNAi. An extensive genetic analysis indicated that unlike mammalian RLRs, *C. elegans drh-1* acts downstream of virus sensing and viRNA biogenesis and that exogenous and antiviral RNAi pathways have distinct genetic requirements. Both the conserved and unique strategies of *C. elegans* in antiviral defense are discussed.

## Results

### Identification of genes required for antiviral silencing in *C. elegans* by feeding RNAi

**Figure 1:**
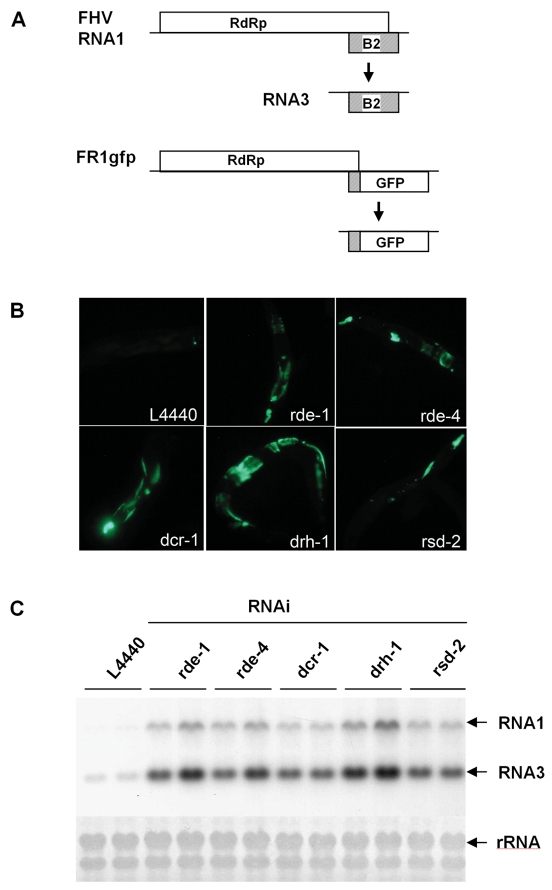
Screening for viral immunity genes in *C. elegans* by feeding RNAi. (A) Genome structure and expression of wildtype FHV RNA1 (FR1) and its B2-deficient mutant, FR1gfp, that expresses the enhanced GFP in place of B2. (B) Detection of green fluorescence in *FR1gfp* reporter worms after feeding RNAi targeting specific genes or the commonly used L4440 vector as indicated, photographed 48 hours after induction of the replicon replication. (C) Accumulation of FR1gfp genomic (RNA1) and subgenomic RNA (RNA3) by northern blotting in *FR1gf* worms with (lanes 3–12) and without (lanes 1–2) feeding RNAi of specific worm genes. Two independent tests were analyzed for each *E. coli* strain. Methylene blue staining of total RNA was provided to show equal loading.

We have previously described a derivative of the infectious full-length cDNA clone of FHV RNA1, pFR1gfp [53], in which eGFP coding sequence replaces most of the VSR B2 coding sequence (Figure 1A). The inserted eGFP fused with the N-terminal 23 codons of B2, is expressed only from the recombinant RNA3 produced during replication of FR1gfp, but not directly from FR1gfp because its initiation codon is more than 2.7 kb away from the 5′-terminus of FR1gfp RNA (Figure 1A). FR1gfp is defective in RNAi suppression due to loss of B2 expression but not in replication so that productive FR1gfp replication and expression of eGFP from the FHV replicon occur only after antiviral RNAi is suppressed by either co-expression of B2 or genetic disruption of the antiviral RNAi pathway in cultured fruit fly and mosquito cells [53]. To develop a model suitable for genetic screens to identify new components in antiviral RNAi, we generated *C. elegans* strains carrying a chromosomally integrated transgene that codes for FR1gfp under the control of a heat-inducible promoter.

**Figure 2:**
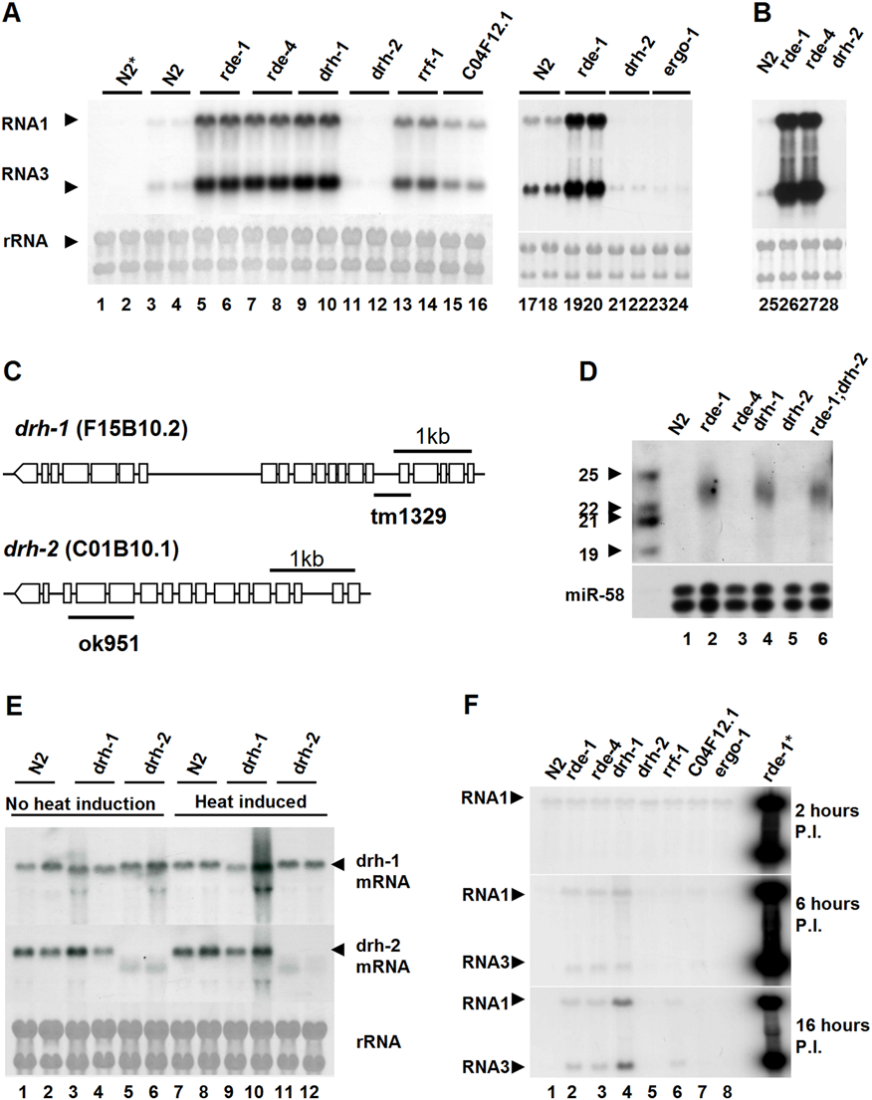
Molecular and functional characterization of the *drh-1* and *drh-2* genes. (A) Accumulation of the FR1gfp replicon RNAs in genetic mutant worm strains carrying the same *FR1gfp* transgene array. Total RNA was also analyzed from wildtype N2 worms with (N2) and without the *FR1gfp* transgene (N2*) 48 hours after induction of the replicon replication (h.a.i.). (B) Induction of the RNAi immunity by the replicon in a worm integrant different from that analyzed in (A). (C) Molecular structures and genetic lesions of the *drh-1* and *drh-2* genes. (D) Accumulation of (-) viral siRNAs in single knockout worm mutants 48 hours after induction of the replicon replication. 45 µg of total small RNAs was loaded in each lane. A combination of 18 ^32^P end-labeled DNA oligos corresponding to eGFP coding sequence in tandem was used as the probe for viral siRNA detection. The same filters were probed for miR-58 after stripping as the loading control. (E) Detection of *drh-1* and *drh-2* transcripts before and after induction of the replicon replication. Two independent tests were analyzed for each strain. (F) Time course analysis of the accumulation of the replicon RNAs in wildtype and mutant worms 2, 6, and 16 h.a.i. Total RNA extracted from *FR1gfp rde-1* worms 48 h.a.i. was loaded as a control (lane rde-1*). Methylene blue staining of total RNA was provided to show equal loading.

We found that no green fluorescence or only a tiny green spot in the pharynx area was observed in FR1gfp worms after heat induction of the FHV replicon transgene (Figure 1B, top left). In contrast, bright green fluorescence was detected throughout the animal body after FR1gfp worms were fed on the *E. coli* food that expresses *rde-1* dsRNA, which depletes mRNA of *rde-1* in a process referred as feeding RNAi (Figure 1B, top middle). Abundant expression of eGFP was also observed in FR1gfp worms after a loss-of-function *rde-1* allele was introduced into FR1gfp worms by genetic crosses (data not shown). Northern blot hybridizations confirmed the abundant accumulation of the chimeric RNA1 and RNA3 in FR1gfp worms after *rde-1* depletion, but FR1gfp replication was inhibited in FR1gfp worms without *rde-1* depletion (Figure 1C, compare lanes 1/2 and 4/5). Productive replication of FR1gfp replicon was similarly rescued by the loss-of-function *rde-1* allele in the second FR1gfp worm strain in which the *FR1gfp* transgene was integrated at a different chromosomal location (Figure 2B, and data not shown). Thus, as found previously in cultured fruit fly and mosquito cells [53], productive FR1gfp replication and detection of extensive eGFP expression in FR1gfp worms depend on the genetic disruption of the antiviral RNAi pathway, suggesting that *FR1gfp* worms could be screened for new components of the pathway by feeding RNAi.

To test this idea, we searched for antiviral RNAi factors among genes shown previously to play a role in exo-RNAi [30],[54]. The genes identified using the candidate gene approach include firstly *dcr-1*, *rde-1*, and *rde-4*, which, together with *rrf-1* and *C04F12.1*, were shown previously to be required for antiviral RNAi against VSV and for exo-RNAi [30],[41],[47],[48]. A specific role of these genes in worm antiviral RNAi was verified by demonstrating genetic rescue of the B2-deficient FHV replicon in FR1gfp worms following introduction of genetic loss-of-function mutations in *rde-1*, *rde-4*, *rrf-1* or *C04F12.1* by genetic crosses (Figure 2A). These results showed that the B2-deficient FHV replicon transcribed from a nuclear transgene was silenced in FR1gfp worms by the same set of genes known to participate in worm antiviral immunity against VSV infection. Thus, the FR1gfp worm strain provided an alternative model to the VSV infection system for the genetic characterization of the RNAi-mediated antiviral immunity in *C. elegans*, including the use of FR1gfp worms for feeding RNAi screens.

In addition to *dcr-1*, *rde-1*, and *rde-4*, 32 of 98 candidate genes tested were required for silencing the viral replicon in *C. elegans* in three independent feeding RNAi screens (Figure 1B/1C, Table S1). Our result suggests that most of the RNAi factors identified from previous genetic and feeding RNAi screens, including *mut-7* and *mut-16* [55], may not contribute to antiviral RNAi. Nevertheless, our screen provided the largest set of putative antiviral RNAi factors in any organism (Table S1), which include *rsd-2* (Figure 1B/1C), required for systemic RNAi [56], and *drh-1*. We focused on *drh-1* identified from the feeding RNAi screen and its related gene *drh-2* because of their homology to the mammalian RLR family of cytosolic sensors for RNA viruses [2],[6],[27].

### *drh-1* is essential for worm antiviral RNAi that is negatively regulated by *drh-2*

*drh-1* encodes one of the three closely related worm members of the RLR family [6],[27]. To verify the result from feeding RNAi, we identified presumptive null alleles of *drh-1(tm1329)* and *drh-2(ok951)* and introduced them respectively into *FR1gfp* worms by genetic crosses (Figure 2C). We did not examine *drh-3* in this study because *drh-3(tm1217)* mutant worms are sterile [27]. We found that *drh-1* mutant worms exhibited no visible morphological and developmental phenotypes. However, *drh-2* worms were noticeably smaller than wildtype worms at the same developmental stages and were late by ∼8 hours in laying eggs (data not shown), suggesting that *drh-2* may have a role in development. Northern blot hybridizations detected mRNAs specific to *drh-1* and *drh-2* in wildtype worms and deletion of 483- and 789-nucleotide genomic DNA respectively in *drh-1* and *drh-2* mutants caused visible shifts of the corresponding mRNAs (Figure 2E). However, neither was transcriptionally induced 48 hours after the initiation of FR1gfp replication or earlier (Figure 2E and data not shown).

We found that RNAs 1 and 3 of the B2-deficient FHV replicon accumulated to high levels in *drh-1* mutant worms as compared to wild-type N2 worms (Figure 2A, compare lanes 3/4 and 9/10). Thus, an essential role for *drh-1* in antiviral silencing revealed by feeding RNAi experiments was confirmed by an independent approach. By comparison, the accumulation levels of the replicon RNAs 1 and 3 were similar in *drh-1*, *rde-1* and *rde-4* worms, but were higher in these worms than in *rrf-1* and *C04F12.1* mutant worms in at least five independent experiments (Figure 2A). These results indicate that antiviral silencing against the FHV replicon was inhibited in *drh-1* worms as effectively as in *rde-1* and *rde-4* mutants, and more effectively than in *rrf-1* and *C04F12.1* mutants (Figure 2A).

**Figure 3:**
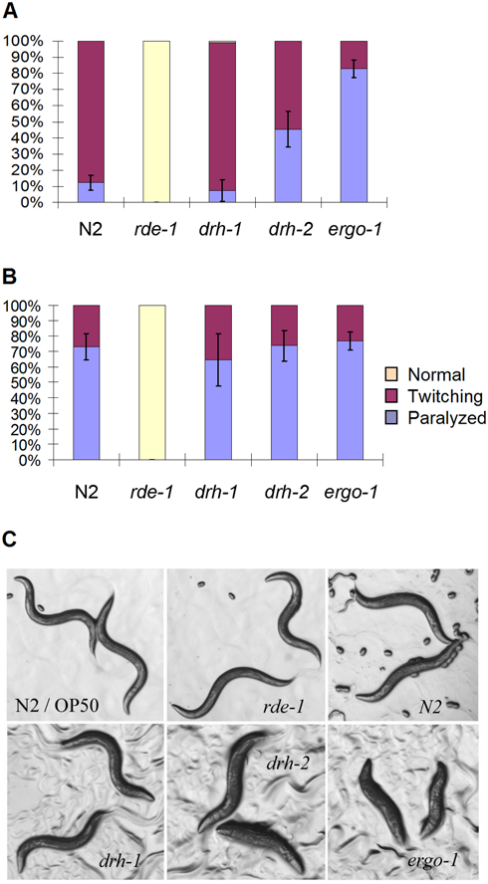
drh-2 is a negative regulator of exogenous RNAi. (A and B) *unc-22* RNAi phenotype in response to unc-22 dsRNA microinjected at 25 and 100 µg/ml, respectively. 30 to 40 worms were used for *unc-22* dsRNA injection. Shown here are the percentages of twitching and paralyzed F1 progenies of each injected worm collected between 8 and 32 hours post injection. The error bars indicate standard deviation for the paralysis phenotype. (C) Morphological phenotype of the F1 progenies of wildtype and mutant worms after feeding RNAi targeting *dpy-13*. All worm strains were synchronized before feeding RNAi.

In contrast, the FHV replicon was not rescued in *drh-2* mutants (Figure 2A, lanes 11–12). Although longer exposure revealed low accumulation levels of the FHV replicon in wild-type worms, the accumulation of the FHV replicon was either undetectable or lower in *drh-2* mutants than in wildtype worms (Figure 2A, compare lanes 17/18 and 21/22). Apparently this effect is not transgenic allele specific in that the same effect was also observed for another FR1gfp transgenic allele (Figure 2B, compare lane 28 and lane 25). As expected, fosmid WRW0640F2, which contains both *drh-1* and *drh-2* wild-type alleles, was able to rescue *drh-1* and *drh*-2 function in the corresponding mutants when assayed for FR1gfp replication (data not shown). These results show that *drh-1* is as important as *rde-1* and *rde-4* in the worm antiviral RNAi against FHV, whereas *drh-2* may negatively regulate this immunity. Similarly reduced accumulation of the FHV replicon was also observed in worms defective for *ergo-1* (Figure 2A, lanes 23 and 24), which encodes an AGO that is required for the biogenesis of endo-siRNAs but is antagonistic to exo-RNAi [41]. Several lines of evidence indicate that *drh-2* and *ergo-1* act as negative regulators of antiviral RNAi rather than positive regulators of FHV replication. FR1gfp is defective in RNAi suppression but not in replication in fly and mosquito cells [53] and in worms because FR1gfp replicated to high levels in several single mutants including *rde-4* (Figure 2A) and robust replication of FR1gfp was not inhibited by the *drh-2* and *ergo-1* alleles in *rde-4*;*drh-2* (see the last section of Results below) and *rde-4*;*ergo-1* (data not shown) double mutants. In addition, both *drh-2* and *ergo-1* negatively regulate exogenous RNAi (Figure 3, see below).

We further performed time course analysis of the accumulation of the FHV replicon in wild-type and seven worm mutants 2, 6 and 16 hours after transcriptional induction of *FR1gfp* (Figure 2F). Primary transcripts of *FR1gfp* initially accumulated to comparable levels in wild-type and all of the seven mutant worm strains examined at two hours after heat induction, indicating that none of these mutant alleles had a major effect on the transcription of the *FR1gfp* locus [30]. Six hours after induction, FHV RNA1 and its replication product RNA3 were detectable in *rde-1*, *rde-4* and *drh-1* mutants, but not in wild-type, *rrf-1*, *C04F12.1*, *drh-2*, or *ergo-1* mutants. At 16 hours after induction, productive replication of the viral replicon also became visible in *rrf-1* worms but not in *C04F12.1*, *drh-2*, or *ergo-1* worms (Figure 2F). These data indicate that the viral RNA clearance was triggered by viral RNA replication in an RNAi-dependent process that might require early and simultaneous participation of DRH-1, RDE-1 and RDE-4.

### Characterization of *drh-1* and *drh-2* mutants in response to exo-RNAi

DRH-1 was first identified as an interacting protein of DCR-1 and subsequent affinity purification coupled with mass spectrometry implicates all three DRH proteins as DCR-1 interactors [6],[27]. *drh-3* mutant worms were defective in germline RNAi but were wild-type in somatic RNAi; however, both germline and somatic RNAi was dramatically reduced in worms treated with either *drh-1* or *drh-2* (or both) dsRNAs [6],[27],[54],[57]. While these data indicate that *drh-3* is essential for germline RNAi, it was uncertain if a specific member of the DRH family plays an essential role in somatic RNAi since depletion of the homologous *drh* genes by dsRNA lacks specificity.

We found that *drh-1* mutant worms, either with or without the *FR1gfp* transgene, remained as sensitive as wild-type worms to feeding RNAi targeting either the maternally expressed *skn-1* (data not shown) or *dpy-13* (Figure 3C), and to microinjection of dsRNA targeting muscle specific gene *unc-22*, which produces both the light (twitching) and severe (paralysis) knockdown phenotypes (Figure 3A/3B). Thus, *drh-1* is dispensable for somatic RNAi, which is in contrast to its essential role in antiviral silencing. Nevertheless, we found a slightly lower percentage of the injected worms exhibiting paralysis in *drh-1* worms than in wildtype worms (Figure 3A/3B), and the difference detected was statistically significant (*P*<0.05), illustrating that *drh-1* worms exhibited a weak deficiency in somatic RNAi. These results suggest that DRH-1 may be more efficient to mediate RNAi in the soma of wild-type worms, but this function of DRH-1 is genetically redundant and could be substituted for by another DRH member in *drh-1* mutant worms.

Surprisingly, *drh-2* mutant worms exhibited enhanced sensitivity to the injection RNAi targeting *unc-22* as compared to wild-type worms and the difference was more obvious when low concentration of dsRNA was injected (Figure 3A/3B). Feeding RNAi targeting *dpy-13* led to mild dumpy phenotype in wild-type and *drh-1* worms, but *drh-2* worms displayed severe dumpy phenotype as did worm mutants known to exhibit an enhanced RNAi phenotype such as *ergo-1* (Figure 3C) and *eri-1* [45],[47],[48]. In contrast to wildtype and *drh-1* worms, *rde-1* worms were resistant to *dpy-13* RNAi as expected (Figure 3C). These results indicate that somatic RNAi is partially suppressed by *drh-2* in wild-type worms, which is similar to its inhibitory role in antiviral RNAi.

### *drh-1* and *drh-2* are dispensable for the biogenesis of miRNAs, endo-siRNAs and piRNAs

**Figure 4:**
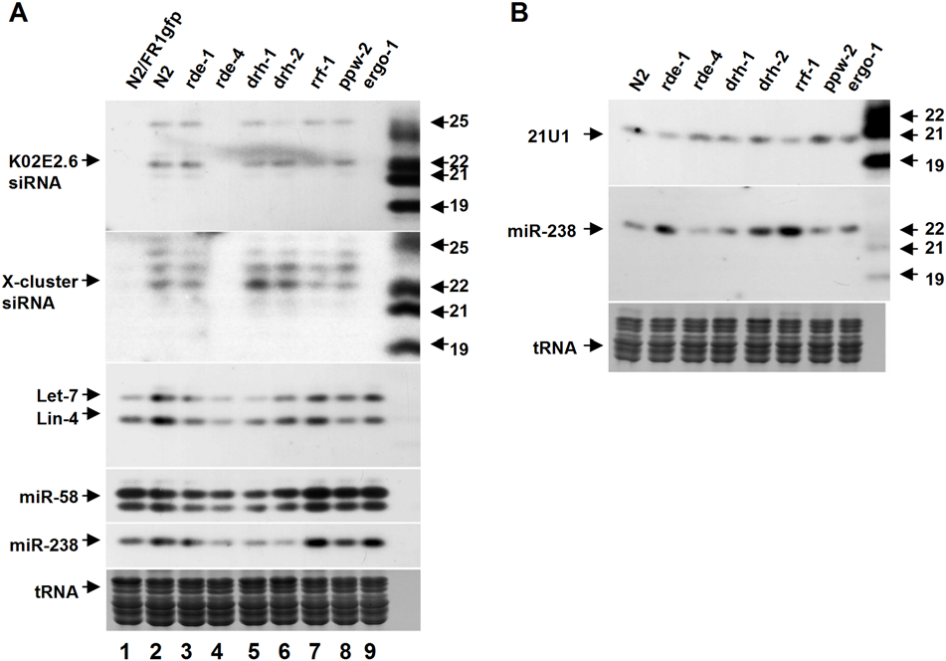
Analysis of endogenous small RNAs. (A) Accumulation of K02E2.6 and X-cluster siRNAs as well as miR-58, miR-238, and let-7 and lin-4 miRNAs in wildtype and mutant worm strains with (lane 1) or without the *FR1gfp* transgene (lanes 2–9). 45 µg of total small RNAs was loaded in each lane. The same filter was repeatedly reprobed after stripping. Small RNAs loaded in lane 1 was extracted from *FR1gfp* worms 48 hours post induction. (B) Accumulation of 21U1 piRNA in wildtype and mutant worm strains in the absence of *FR1gfp* transgene. End-labeled DNA oligos complementary to endo-siRNAs, miRNA and 21U1 piRNA were used as the probes. Ethidium bromide staining of tRNAs was provided to show equal loading.

*drh-3* is required for the biogenesis of endo-siRNAs, but not for miRNAs or piRNAs [27],[57]. Production of endo-siRNAs is also dependent on *ergo-1*, which inhibits both somatic RNAi and antiviral silencing. To investigate the mechanism of *drh-1* and *drh-2*, we further determined if either was involved in the biogenesis of the three classes of *C. elegans* small RNAs. We found that neither *drh-1* nor *drh-2* was required for the biogenesis of miRNAs (Figure 4A, lanes 5 and 6), which is also known to be independent of *rde-4*. Minor changes in the accumulation of miRNAs observed occasionally in *drh-1* mutant worms were not reproducible (Figure 2D/3A/3B/5B). The *rde-4*-dependent endo-siRNAs K02E2.6 and X-cluster siRNAs also accumulated to wild-type levels in both *drh-1* and *drh-2* mutants (Figure 4A/4B). The recently characterized piRNAs also accumulated to wild-type levels in both *drh-1* and *drh-2* mutants as in *rde-4*, *rde-1*, *rrf-1*, *ppw-2* and *ergo-1* mutants (Figure 4B). Thus, *drh-1* and *drh-2* do not appear to contribute to the biogenesis of any of the known endogenous small RNAs in *C. elegans*, suggesting that *drh-2* negatively regulates somatic RNAi and antiviral silencing in a mechanism distinct from *ergo-1*.

### *drh-1* acts downstream of *rde-4*-dependent production of viRNAs

**Figure 5:**
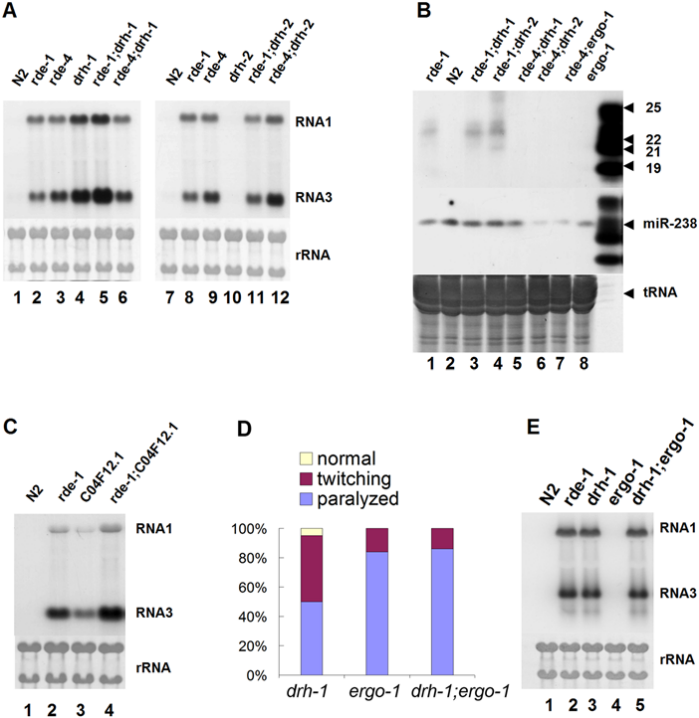
Genetic epistatic analysis of antiviral RNAi. (A, C, E) Accumulation of the FR1gfp replicon RNAs in single and double knockout worm mutants 48 after replicon replication. (B) Accumulation of (-) viRNAs in single and double knockout worm mutants 48 after replicon replication as described in Figure 2. A combination of 18 ^32^P end-labeled DNA oligos corresponding to eGFP coding sequence in tandem was used as the probe for viral siRNA detection. The same filters were probed for miR-238 after stripping. (D) *unc-22* RNAi phenotype in response to unc-22 dsRNA microinjected at 100 µg/ml as described in Figure 3.

An antiviral RNAi component may function in virus sensing, the biogenesis or the antiviral activity of viRNAs [4]. FHV-specific viRNAs of the antigenomic polarity, (-)viRNAs, were detectable in both *drh-1* and *rde-1* mutants (Figure 2D, lanes 2 and 4). Probing for viRNAs of the genomic polarity resulted in a smear and no discrete bands were detected in any of the worm strains tested either before or after transcriptional induction of *FR1gfp* (data not shown). FHV viRNAs accumulated to much lower levels in worms than in fruit flies [13],[23]. ViRNAs in worm cells infected with VSV were only detected by the RNase protection assay [47], which is far more sensitive than Northern blot hybridization [58]. We found that (-)viRNAs were undetectable in wild-type, *drh-2* or *ergo-1* mutant worms (Figure 2D, lanes 1 and 5; Figure 5B, lane 8), which is likely due to the inhibition of the viral replication (Figure 2A, lanes 3/4, 11/12, 23/24) and consequently lower levels of viral dsRNA for dicing in these worms. The FHV replicon replicated to similarly high levels in *rde-1*, *rde-4* and *drh-1* mutant worms (Figure 2A), but viRNAs were not detectable in *rde-4* worms, unlike in *rde-1* and *drh-1* worms (Figure 2E). These results therefore show that RDE-4 is essential for the production of FHV viRNAs whereas either DRH-1 or RDE-1 is dispensable. However, since viRNAs produced in *drh-1* and *rde-1* worms were not able to inhibit the replication of the VSR-deficient viral replicon (Figure 2A), we further conclude that both RDE-1 and DRH-1 are required for the antiviral activity of viRNAs. The observations that the viral RNA trigger was detected and processed into viRNAs in *rde-1*and *drh-1* mutant worms ruled out a direct role of either gene in virus sensing.

### Characterization of antiviral silencing in double mutants

To determine the epistatic relationships among the identified antiviral RNAi components, we constructed seven double knockout worm mutants by genetic crosses. First, rescue of the VSR-deficient viral replicon was stronger in *rde-1* worms than in the mutant worms defective for *C04F12.1* (Figure 5C), which encodes an AGO closely related to *rde-1* in the expanded group of AGOs specific to *C. elegans*. However, the viral replicon replicated to higher levels in the *rde-1*:*C04F12.1* double mutant than in either single mutant (Figure 5C, compare lane 4 and lanes 2 and 3). Thus, there is an additive effect of the two mutant alleles in blocking antiviral RNAi, indicating parallel AGO pathways for antiviral silencing in *C. elegans*. Second, the viral replicon did not accumulate to higher levels in *rde-4*;*drh-1* double mutant than in either *rde-4* or *drh-1* single mutant (Figure 5A, compare lane 6 and lanes 3 and 4). viRNAs were not detectable in *rde-4*;*drh-1* worms (Figure 5B, lane 5), which was similar to *rde-4* worms but distinct to *drh-1* worms. These results illustrated that *rde-4* and *drh-1* act in the same antiviral RNAi pathway and placed *rde-4* in the upstream of *drh-*1. Third, abundant viRNAs were detected in *rde-1*;*drh-1* worms as was found in *rde-1* and *drh-1* single mutants, further confirming the above conclusion that neither gene is essential for viRNA production.

Fourth, we found that the viral replicon replicated to similar levels in *rde-1*, *rde-4* single mutants and *rde-1*;*drh-2* and *rde-4*;*drh-2* double mutants (Figure 5A, compare lanes 11 and 12 with lanes 8 and 9). This result showed that the *drh-2* mutant allele failed to enhance antiviral RNAi when either *rde-4* or *rde-1* was not functional, suggesting that the *rde-4*-initiated pathway was targeted by *drh-2* for negative regulation. Fifth, the accumulation levels of the viral replicon were similar in the *ergo-1*;*drh-1* double mutant and the *drh-1* single mutant (Figure 5E), indicating that the *ergo-1* mutant allele also became ineffective in enhancing antiviral RNAi when *rde-4*-initiated pathway was inactive. However, we found that the *ergo-1*;*drh-1* double mutant exhibited enhanced sensitivity to somatic RNAi as did the *ergo-1* single mutant (Figure 5D), further demonstrating that *drh-1* was dispensable for exo-RNAi.

## Discussion

Recent studies demonstrate that viruses can infect, replicate, and assemble within *C. elegans* cells and that RNA viruses are targeted for silencing by the canonical RNAi pathway in *C. elegans* [46]–[49]. These findings suggest that *C. elegans* could become an important model for understanding basic aspects of virus–host interactions [59]. In this work, we developed a *C. elegans* model for the genetic analysis of antiviral RNAi that exhibits several notable features. Our system is amenable to the powerful genetic tools in *C. elegans* since initiation of viral replication is inducible without the need for viral inoculation. The viral replication cycle in our system begins with translation of the viral RdRP in the cytosol from nuclear transcripts and bypasses the initial steps in virus entry during infection such as infection of worm cells by VSV. However, all of the five *C. elegans* genes known to participate in antiviral RNAi immunity against VSV infection were also required for inhibiting the replication of the FHV replicon. In addition, mutant alleles of genes in the endo-RNAi pathway that enhance exoRNAi response also increased potency of antiviral RNAi against both VSV (*eri-1* and *rrf-3*) [47],[48] and the FHV replicon (*ergo-1*). These data indicate that antiviral RNAi is triggered during intracellular viral RNA replication in the absence of receptor-mediated virus entry across the plasma membrane as is known for viral immunity initiated by mammalian RLRs. Furthermore, removal of VSR B2 enhances the sensitivity of the GFP-expressing FHV replicon to antiviral RNAi. This is in contrast to the use of VSR-expressing wild-type viruses in previous genetic analyses [16],[60], which may explain why our pilot feeding RNAi screen led to the identification of a large set of putative antiviral RNAi factors.

### Genetic requirements of antiviral RNAi in *C. elegans*

Our genetic analysis confirms earlier observations that antiviral RNAi in *C. elegans* overlaps the canonical dsRNA-siRNA pathway of RNAi [46]–[48]. For example, 35 known RNAi factors participate in the worm antiviral RNAi against FHV (Table S1). Antiviral RNAi against FHV is enhanced in both *ergo-1* and *drh-2* mutant worms that exhibit enhanced RNAi. Furthermore, this work together with previous studies shows that (i) antiviral RNAi induced by either FHV or VSV requires *dcr-1*, (ii) production of both VSV and FHV siRNAs was dependent on *rde-4* and (iii) neither *rde-1* nor *drh-1* is essential for the biogenesis of viRNAs. These findings revealed a role of RDE-4, most likely with DCR-1 in a previously identified complex, in the sensing of viral RNA trigger and in the biogenesis of viRNAs. Whereas these data support a shared biogenesis pathway for viRNAs, exo- and endo-siRNAs in *C. elegans* [30], an indispensable role for *rde-4* in the viRNA biogenesis is distinct from *D. melanogaster* in which *DCR-2* but not the *rde-4* homolog (*R2D2*), is essential for viRNA production [14].

Primary siRNAs processed directly from exogenous dsRNA are not sufficient abundant to detect by Northern blot hybridizations in *rde-1* worms [35],[43],[58], in contrast to FHV viRNAs. This difference is probably due to the robust supply of viral dsRNA from active viral RNA replication in *rde-1* worms whereas the amount of exogenous dsRNA is limited in the absence of RDR-dependent dsRNA synthesis. Furthermore, our feeding RNAi screens indicate that a majority of the known exoRNAi factors may not participate in the worm antiviral RNAi against FHV (Table S1). In addition, whereas *drh-1* is essential for antiviral RNAi against FHV, it is dispensable for exoRNAi. These data suggest that antiviral RNAi and exoRNAi pathways in *C. elegans* are genetically distinct even though both are initiated by RDE-4 and DCR-1.

An increased inhibition of the worm antiviral RNAi against FHV was observed in *rde-1*;*C04F12.1* double mutant than in either single mutant, indicating that FHV is targeted by two parallel AGO-dependent antiviral RNAi pathways in *C. elegans*. This conclusion is also supported by previously observations that knockdown of either *rde-1* or *C04F12.1* individually enhances the VSV accumulation [48] and that RNAi suppression by B2 further increases FHV accumulation in *rde-1* worms [46]. Both RDE-1 and C04F12.1 belong to the substantially expanded subfamily of AGOs found only in worms and many members in this subfamily have been shown to act either in parallel or sequentially in exo-RNAi [41],[42]. Thus, expansion of this AGO subfamily in *C. elegans* may represent a unique strategy of host adaptation to viral infection, distinct or in addition to the strategy used by insects in which the evolution rate of antiviral RNAi factors DCR2 and AGO2 is much faster than that of their miRNA pathway counterparts DCR1 and AGO1 [61],[62].

### DRH-1 directs antiviral RNAi downstream of viRNA biogenesis

Several lines of evidence illustrate that *drh-1* has a specific, non-redundant role in the *rde-4*-dependent antiviral RNAi pathway in *C. elegans*. Either depletion of *drh-1* mRNA by feeding RNAi or a genetic lesion in *drh-1* blocked antiviral RNAi against FR1gfp, an FHV-based replicon that does not express VSR B2 and thus exhibits a specific defect to suppress antiviral RNAi. B2 is a dsRNA/siRNA-binding protein which is located both inside the viral replication complex to inhibit the dicing of nascent dsRNA replicative intermediates into viRNAs and in the cytoplasm to inhibit the activity of viral siRNAs [13],[23],[46],[63],[64]. The antiviral RNAi against FHV was inhibited in *drh-1* worms as efficiently as in *rde-4* and *rde-1* mutant worms. A time course analysis further suggests that DRH-1, RDE-4 and RDE-1 may all participate in the early induction of antiviral RNAi. Finally, epistatic analysis showed that the FHV replicon did not replicate to higher levels in *rde-4*:*drh-1* double mutant than in either single mutant, demonstrating that *drh-1* specifically acts in the *rde-4*-initiated antiviral RNAi pathway. Notably, inhibition of antiviral RNAi by the *drh-1* mutant allele did not prevent the production of viRNAs in either *drh-1* single mutant or *rde-1*:*drh-1* double mutant worms, indicating that defects of *drh-1* worms in antiviral RNAi were similar to *rde-1* mutants but distinct to *rde-4* worms. These data together show that *drh-1* of *C. elegans* acts downstream of *dcr-1* and *rde-4* in the antiviral RNAi pathway and does not play a critical role in the sensing of the viral RNA trigger.

### *C. elegans* DRHs may regulate specificity of distinct siRNA pathways

Previous knockdown experiments have established a genetic requirement for the DRH family of genes in the canonical RNAi [6],[27]. Use of *drh-3* mutant worms has further shown that DRH-3 mediates germline RNAi and endo-RNAi, but is dispensable for somatic RNAi [27],[57]. Our results show that neither *drh-1* nor *drh-2* plays a role in the biogenesis of miRNAs, endo-siRNAs and piRNAs and that *drh-1* mutant worms supported RNAi in the soma with only a negligibly reduced efficiency. Thus, *drh-1* is essential for antiviral RNAi but is largely dispensable for both endo-RNAi and somatic RNAi. In contrast, *drh-2* mutant worms exhibited enhanced response to both somatic RNAi and antiviral RNAi. Isolation of double *drh* knockout mutants, which is not possible to achieve by genetic crosses between the single mutants because of their close proximity, will be necessary to determine if the observed somatic RNAi in *drh-1* worms is mediated by *drh-3* and/or *drh-2*.

Current models indicate that DRH-1/2 and DRH-3 participate in the biogenesis of exo-and endo-siRNAs, respectively, in distinct complexes with DCR-1 and that primary siRNAs thus generated are loaded in a particular AGO to guide RDR-dependent amplification of secondary siRNAs required for specific degradation of target mRNA [27],[34],[41],[58],[65]. Results presented in this work indicate that DRH family proteins play an essential role in siRNA pathways by acting downstream of the DCR-1/RDE-4-dependent siRNA biogenesis to mediate both specific and redundant siRNA pathways. Since in vivo interactions of DRH proteins with DCR-1 and RDE-4 have been detected [6],[27], we propose that DRH protein facilitate the binding of primary siRNAs to AGOs, the amplification of secondary siRNAs, or the targeting and cleavages of the mRNA. The essential role of DRH-3 and DRH-1 specifically in the endo-siRNA/germline exo-siRNA and viRNA pathways, respectively, may be due to tissue and cell-specific localization of DRH proteins and of dsRNA trigger and mRNA targets. In contrast, somatic RNAi may be mediated redundantly by DRH-1, possibly with DRH-3, which is supported indirectly by the observation that although all of the known endo-RNAi worm mutants are defective in the biogenesis of endo-siRNAs, enhancement of somatic RNAi were detected in *rrf-3*, *eri-1* and *ergo-1* mutants, but was not reported for *drh-3* mutant worms. DRH-2 may compete with DRH-1 for binding to the same or a similar set of co-factors, thereby inhibiting DRH-1-dependent antiviral RNAi and somatic RNAi. This hypothesis is consistent with our observation that the *drh-2* mutant allele failed to enhance antiviral RNAi against FHV in *rde-1* and *rde-4* mutant worms that are defective in the *drh-1*-dependent viRNA pathway.

### Conserved antiviral function of RLR members in *C. elegans* and mammals

Our demonstration that the viral immunity is regulated by *drh-1* and *drh-2* in *C. elegans* indicates an evolutionarily conserved antiviral role of the RLR family between worms and mammals. In mammals, RLR members RIG-I and MDA5 are essential for controlling infection of two distinct sets of ssRNA viruses. Current models envision that recognition of specific viral RNA forms by the C-terminal repressor domain activates a signaling cascade via two caspase activation and recruitment domains (CARD) at the N-terminus that culminates in the transcription of cytokine genes and broad-spectrum immunity [1]–[3]. The third RLR member LGP2 is analogous to DRH-2 since it shares homology with RIG-I and MDA5 in the helicase and repressor domains without the N-terminal CARD domains, and appears to repress RIG-I signaling but contribute to MAD5 signaling [1]–[3]. Although the N-terminal regions of DRH-1 and DRH-3 are highly homologous, neither contains a CARD domain or is transcriptionally induced upon viral replication. Our genetic analysis further suggests that *drh-1* directs antiviral RNAi downstream of both the sensing and the processing of the viral RNA trigger into viRNAs. This may explain why DRH proteins of *C. elegans* do not contain CARD domains, which mediate downstream signaling events by protein-protein interactions in mammals.

## Methods

### *C. elegans* genetics and culture

The Bristol strain N2 was used as the standard wild-type strain. Alleles used in this study are all derived from N2 and include *rde-1(ne300)*, *rde-4(ne337)*, *drh-1(tm1329)*, *drh-2(ok951)*, *rrf-1(pk1417)*, *ergo-1(tm1860)*, and *C04F12.1(tm1637)*. The genotypes of *rde-1* and *rde-4* worms were confirmed using *skn-1* feeding RNAi. The genotypes of the rest worm strains containing single or double mutations were confirmed by PCR and/or feeding RNAi targeting *skn-1*.

### Transgene construct and transgenic worms

Transgene construct carrying FR1gfp replicon was a derivative of pFR1-3 by replacing the NcoI-SacI fragment of FHV RNA1 by the full length enhanced GFP coding region as described previously [46],[53]. This created a translational fusion of GFP with the N-terminal 23 amino acids of B2 and deletion of approximately 200 nucleotides from the B2 ORF. Animals were made transgenic by gonadal microinjection following standard protocol as described [46]. Briefly, FR1gfp plasmid (final concentration 5 µg/µl) was mixed with the rol-6D plasmid pRF4 (final concentration 100 µg/µl) for injection into wild-type N2 animals. Generation of worm integrants carrying FR1gfp transgene and assay for viral replication was carried out as described previously [46].

### RNAi experiments

Injection and feeding RNAi were carried out as previously reported. Briefly, for *unc-22* dsRNA preparation, *unc-22* single-stranded RNAs of both polarities were *in vitro* transcribed from a cDNA fragment amplified using T7 promoter-tagged primer unc22T7plus (TAATACGACTCACTATAGGAGTTGGGAGAGGATGAAGCT) and primer unc22T7minus (TAATACGACTCACTATAGGCCACCGTTGTCACGTGGAGGA). For injection RNAi, *unc-22* dsRNA at 25 µg/ml or 100 µg/ml in water was delivered into intestine of young adult worms through microinjection. The injected worms were then transferred onto fresh NGM plates 8 hours post microinjection. Progenies produced between 8 and 32 hours post microinjection were scored for *unc-22* phenotype. Feeding RNAi targeting *skn-1* or *dpy-13* was performed by feeding worms on *E. coli*. HT115 strains that express dsRNA corresponding to *skn-1* and *dpy-13*, respectively. IPTG at final concentration of 1 mM was used for the induction of dsRNA expression. The P-value on the differences of *unc-22* RNAi phenotype (paralysis) between worm strains was calculated using unpaired t test calculator (http://www.graphpad.com/quickcalcs/ttest1.cfm?FormatSD).

### RNA preparation and Northern analysis

Total RNA was prepared using the TRI Reagent method (MRC, Inc.). Small RNAs were enriched using the mirVana kit (Ambion). For high molecular viral RNA analysis, 3 to 6 µg total RNA per lane was fractionated in 1.2% agarose gel. For small RNA analysis, 30 to 50 µg of enriched small RNAs per lane was resolved using 15% acrylamide denaturing gel along with chemically synthesized and end-labeled siRNAs as size markers. After electrophoreses, the RNA samples were transferred onto Hybond N+ membrane (Amersham Biosciences) and UV crosslinked using 1.8×10^5^ µJ/CM^2^ as output power (SpectroLinker). For small RNA analysis, membranes were hybridized with ^32^P-labeled oligo DNA probes in PerfectHyb buffer (Sigma). For northern blot detection of *drh-1* and *drh-2* transcripts, ^32^P-labelled DNA probes were prepared using genomic DNA fragments amplified by PCR. Primer tm1329_internal_b (ATACTCTGCCTCGAGCCGAT) and primer Tm1329minus (TCAGTCGTATCTCCAATTTTCGA) were used to amplify genomic DNA as the *drh-1* specific probe, while primer drh-21780plus (AGTAGCATTCGTTCGAGAGTT) and primer Ok951minus (TTGCTTTCCTGGACATGAAGTG) were used to generate the *drh-2* specific probe. Sequences for oligo probes used for the detection of endogenous small RNAs were: miR-238, CTGAATGGCATCGGAGTACAAA; miR-58, ATTGCCGTACTGAACGATCTCA; miR-2, GCACATCAAAGCTGGCTGTGATA; lin-4, TCACACTTGAGGTCTCAGGGA; Let-7, AACTATACAACCTACTACCTCA; X-cluster siRNA, CGCGTATCTATTCAATTGAAT; K02E2.6 siRNA, ATCAGTTACTTGCCAATTTC; and 21U-1, CACGGTTAACGTACGTACCA

### *drh-1* and *drh-2* functional rescue experiments

Transgenic lines in either *drh-1(tm1329)* or *drh-2(ok951)* background that carried an extrachromosomal array corresponding to WRW0640F2 were produced with microinjection, and were crossed respectively with *drh-1(tm1329)* and *drh-2(ok951)* animals homozygous for *FR1gfp* transgene. The F1 progenies in either *drh-1(tm1329)* or *drh-2(ok951)* background were then examined for GFP expression after being maintained at 20°C (*drh-1*) or 25°C (*drh-2*) for 36 hours after induction of the FR1gfp replicon replication. The complete loss or significant reduction of green fluorescence in F1 progenies that carried both *FR1gfp* and the WRW0640F2 extrachromosomal array, as compared to that in F1 animals that carried *FR1gfp* only, was considered as successful *drh-1* function rescue. Likewise, increased GFP expression in F1 progenies that carried *FR1gfp* transgene only as compared to F1 progenies that carried both *FR1gfp* transgene and a WRW0640F2 extrachromosomal array, was considered as evidence for the restoration of *drh-2* function.

### Imaging microscopy

GFP fluorescence images were collected using a CANON G2 digital camera mounted on an Olympus IMT-2 microscope.

## Supporting Information

Table S1 Putative antiviral RNAi factors identified by feeding RNAi screens
(0.06 MB DOC)
