# Supplementary material for: An RIG-I-Like RNA Helicase Mediates Antiviral RNAi Downstream of Viral siRNA Biogenesis in Caenorhabditis elegans
Source: PLoS Pathog. 2009 Feb 6;5(2):e1000286. doi: 10.1371/journal.ppat.1000286 (PMC2629121; doi:10.1371/journal.ppat.1000286)
Supplement: Table S1 — Putative antiviral RNAi factors identified by feeding RNAi screens (0.06 MB DOC) [file ppat.1000286.s001.doc]

**Supplemental Table 1. Putative antiviral RNAi factors identified by feeding RNAi screens**

| **Genes targeted** | **locus** | **description** | **eGFP score*** |
| --- | --- | --- | --- |
| **Known RNAi genes** | | | |
| K12H4.8 | *dcr-1* | PAZ, RNase III, dsRNA binding | +++(viable) |
| K08H10.7 | *rde-1* | PAZ and PIWI domain protein | +++(viable) |
| T20G5.11 | *rde-4* | dsRNA binding | +++(viable) |
| F15B10.2 | *drh-1* | DEAD/DEAH box RNA helicase | +++(viable) |
| F52G2.2 | *rsd-2* | Systemic RNAi | ++(viable) |
| Y48G8AL6 | *smg-2* | NMD protein, RNAi persistence | ++(viable) |
| **Chromatin factors** | | | |
| M03C11.3 |  | Chromatin associated protein (*VC764*-0k1265) | +++ (viable) |
| K07A1.11 | *rba-1* | Rb-binding protein;CAF-1 like | +++ |
| ZK1127.7 |  | DNA topoisomerase type II | ++ |
| **Nuclear import/export factors** | | | |
| F32E10.4 | *ima-3* | Importin-α family | +++ |
| F59A2.1 | *npp-9* | Ran GTPase-binding protein | ++ |
| **RNA binding & processing** | | | |
| K08D10.4 | *rnp-2* | Splicesomal protein | ++ (viable) |
| W05H7.4 |  | Zn-finger protein | ++ (viable) |
| E02H1.1 |  | Ribosomal RNA adenine dimethylase | ++ |
| F26A3.2 |  | Nuclear cap-binding protein complex | ++ |
| F49D11.1 |  | Pre-mRNA splicing factor PRP17 | ++ |
| T25G3.3 |  | Upf1p-interacting protein in yeast | ++ |
| ZK1127.5 |  | RNA 3’-terminal phosphate cyclase | ++ |
| **DEAD/DEAH helicase domains** | | | |
| F56D2.6 |  | DEAD/DEAH-box RNA helicase | ++ |
| **Transcription** | | | |
| ZK1127.6,9 |  | Transcription elongation factor CA150 | ++ (viable) |
| W10C8.2 | *pop-1* | Transcription factor TCF-4 | ++ |
| C16A3.4 |  | C2H2-type Zn-finger domain | +++ |
| C55B7.5 |  | Prefoldin chaperone domain | +++ |
| W06E11.1 |  | RNA polymerase III subunit | ++ |
| **Signaling** | | | |
| T01G9.6 | *kin-10* | Casein kinase II,beta subunit | +++ |
| **Other** | | | |
| F54H12.1 | *aco-2* | Mitochondrial aconitase homolog | ++ |
| T09A5.10 | *lin-5* | Spindle apparatus component | ++ |
| F56A3.4 | *spd-5* | Mitotic spindle assebly | ++ |
| W04C9.1 | *haf-4* | ABC transporter | ++ |
| **Unknown function** | | | |
| C06A5.1 |  |  | ++ |
| C29E4.2 |  |  | +++ |
| F26E4.4 |  |  | ++ |
| T23D8.3 |  |  | ++ |
| W04A4.5 |  |  | +++ |
| F37B12.4 | Tm1052  Tm1245 | Ubiquitin carboxyl-terminal hydrolase | ++(viable) |

*Three independent screens were performed under the same conditions. Feeding RNAi targeting *rde-1* served as positive control. Feeding RNAi targeting no gene (empty vector) served as negative control. Genes produced GFP fluorescence with strength as that found for *rde-1* worms during feeding RNAi screen were scored as +++ candidates, whereas genes produced GFP fluorescence weaker than that in *rde-1* feeding RNAi but significantly stronger than that in non-targeting worms were score as ++ candidates.
